# Regulation of Renal and Extrarenal Calcitriol Synthesis and Its Clinical Implications

**DOI:** 10.3390/ijms26125570

**Published:** 2025-06-11

**Authors:** Armin Zittermann

**Affiliations:** Clinic for Thoracic and Cardiovascular Surgery, Heart & Diabetes Center North Rhine-Westphalia, Ruhr University Bochum, 32545 Bad Oeynhausen, Germany; azittermann@hdz-nrw.de; Tel.: +49-5731-97-0

**Keywords:** calcitriol, 1,25-dihydroxyvitamin D, vitamin D supplementation, calcium, phosphate, phosphorus, parathyroid hormone, fibroblast growth factor-23, meta-analysis, 25-hydroxyvitamin D

## Abstract

There is evidence that calcitriol is the only biologically active vitamin D metabolite. This review summarizes data on the regulation of renal and extrarenal synthesis of calcitriol by nutritional, physiologic, mechanical, genetic, and disease-related factors. Relatively low circulating calcitriol due to low substrate availability, i.e., low circulating 25-hydroxyvitamin D, has been reported in nutritional rickets, osteomalacia, obesity, and preeclampsia. In these situations, vitamin D supplementation can increase circulating calcitriol and, together with calcium, prevent rickets/osteomalacia and reduce the risk of preeclampsia and obesity-related type 2 diabetes mellitus. However, the correction of low circulating calcitriol due to mechanical unloading/immobilization by vitamin D supplementation is not effective in preventing osteoporotic fractures. Circulating calcitriol is also low in diseases such as cardiac and renal failure. Both illnesses share some other similarities regarding dysregulated calcium/phosphate metabolism, including elevated parathyroid hormone and fibroblast growth factor-23, suggesting similar treatment strategies. Genetic disorders of vitamin D metabolism are rare and can affect circulating calcitriol differently. Calcitriol synthesis in immune cells is obviously not primarily dependent on circulating 25-hydroxyvitamin D, which challenges the use of vitamin D for infection prevention. Since various factors can differently influence calcitriol regulation, more personalized preventive/therapeutic strategies of targeting calcitriol synthesis are necessary.

## 1. Introduction

**Figure 1:**
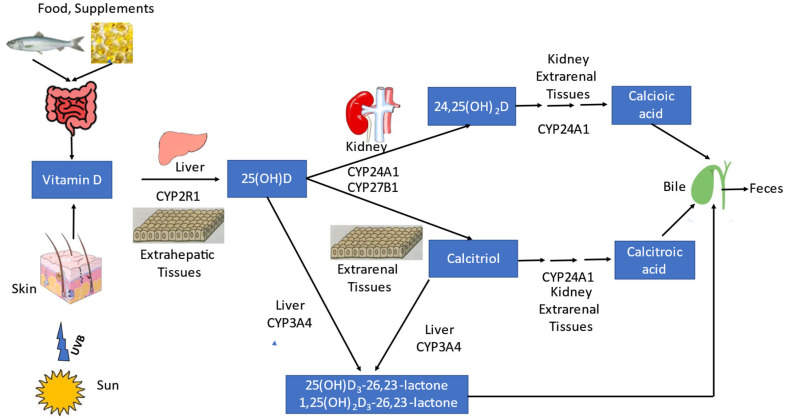
Vitamin D metabolism in the human body. Figure legend: Vitamin D is produced by skin synthesis after solar ultraviolet B (290–315 nm) irradiation or ingestion by food or supplements. Thereafter, it is hydroxylated in the liver and extrahepatic tissues by CYP2R1 to 25-hydroxyvitamin D (25[OH]D), and in the kidney and extrarenal tissues by CYP27B1 to the vitamin D hormone 1,25-dihydroxyvitamin D (calcitriol). Degradation of calcitriol and 25(OH)D is performed by CYP24A1 via several intermediate products to calcitroic acid and calcioic acid, respectively, which are both excreted via bile in the feces. Calcitriol and 25(OH)D can also be degraded by hepatic CYP3A4 to the respective 26,23-lactones, which are also excreted via bile in the feces.

Circulating 25-hydroxyvitamin D (25[OH]D; when written without the D_2_ or D_3_ subscript, either/or both forms of vitamin D are meant) is the generally accepted indicator of human vitamin D status [1]. It reflects the sum of skin synthesis of vitamin D, dietary vitamin D intake, and vitamin D supplement use (Figure 1). It has a relatively long half-life of several weeks to months [2,3], thus indicating mid-term vitamin D status. According to the Institute of Medicine, circulating 25(OH)D < 30 nmol/L and between 30 and 50 nmol/L have been classified as deficient and inadequate, respectively [4]. However, 25(OH)D in itself is not directly biologically active, but is further metabolized by a 1α-hydroxylase (CYP27B1) to 1,25-dihydroxycholecalciferol, also designated calcitriol [5]. Earlier studies have demonstrated the exclusive physiological relevance of calcitriol since administration of 24,24-difluoro-25-hydroxyvitamin D_3_, a metabolite that cannot be hydroxylated at the C_24_ position, was not associated with any deficiency in experimental animals for two generations. Although the C_24_ position is important for the metabolism of 25(OH)D and calcitriol, the animals were fully able to carry out reproduction and development [6,7], indicating that 24-hydroxylation induces only vitamin D catabolism. Likewise, vitamin D administration resulted in a rapid increase in circulating calcitriol in patients with osteomalacia, while circulating 24,25- and 25,26-dihydroxycholecalciferols rose only gradually, after circulating 25(OH)D concentration had increased to normal. This indicated that hydroxylated vitamin D metabolites other than calcitriol are unimportant [8].

It has long been assumed that the measurement of circulating calcitriol is neither necessary or not helpful because of the homeostatic regulation of its blood concentration [1]. In addition, calcitriol was a difficult analyte to measure, due to its low blood concentration [9]. Even more important is the argument that measures circulating 25(OH)D also reflects circulating calcitriol homeostasis since deficient or insufficient circulating 25(OH)D concentrations are dose-dependently associated with elevated concentrations of parathyroid hormone (PTH) [10], an inducer of renal calcitriol synthesis. In line with this, a positive association has been reported between vitamin D supplementation and circulating calcitriol, at least in individuals with baseline 25(OH)D < 50 nmol/L [11]. Likewise, in individuals with and without osteomalacia receiving radioactive-labeled vitamin D, formation of radioactive calcitriol was detectable only in vitamin D-deficient subjects [12].

Despite the association of circulating 25(OH)D with calcitriol homeostasis, there is accumulating evidence that substrate availability is not the only factor influencing circulating calcitriol homeostasis (see below). Since calcitriol is an important steroid hormone, this review was aimed at providing a comprehensive overview regarding the regulation of circulating calcitriol by nutritional factors, mechanical loading/unloading, genetic disorders, and specific diseases. Meta-analysis (MA) is performed if the impact of a factor is uncertain. Only controlled trials including a control period or control group are considered. In addition, tissue synthesis and regulation of calcitriol are appraised. Finally, clinical implications are discussed.

## 2. Regulation of Circulating Calcitriol, Calcium, and Phosphorus

### 2.1. Calcitriol Metabolism

**Figure 2:**
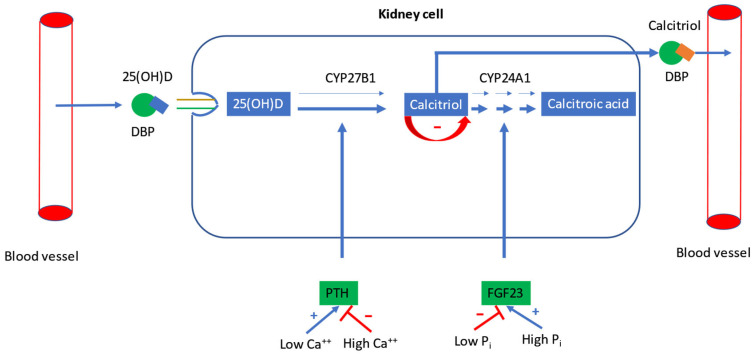
Regulation of calcitriol synthesis in kidney cells. Figure’s legend: In the circulation, 25(OH)D is mainly bound to DBP. DBP-bound 25(OH)D enters the renal cell via megalin-dependent, cubilin-mediated endocytosis. It is then metabolized by CYP27B1 to calcitriol. Calcitriol is released into the bloodstream, where it is mainly transported bound to DBP. Calcitriol biosynthesis is increased by low serum ionized Ca via PTH, and is suppressed by high serum ionized Ca via down-regulation of PTH. High serum P_i_ stimulates FGF23, which increases degradation of calcitriol to calcitroic acid by CYP24A1. Hypophosphatemia suppresses FGF23 synthesis. By a negative feedback mechanism, calcitriol can suppress its own synthesis. In CKD, hyperphosphatemia leads to increased FGF23, which results in suppressed calcitriol biosynthesis in proximal tubular cells. Subsequently, the intestinal calcium absorption rate decreases, which leads to stimulation of PTH secretion. Thin arrows indicate loss-of-function mutations. Abbreviations: 25(OH)D, 25-hydroxyvitamin D; DBP, vitamin D binding protein; PTH, parathyroid hormone; FGF23, fibroblast growth factor 23; Ca, calcium; P_i_, inorganic phosphate; CKD, chronic kidney disease; +, stimulation; −, suppression.

The synthesis of calcitriol primarily takes place in the kidneys, after uptake of DBP (vitamin D-binding protein)-bound circulating 25(OH)D by megalin-dependent cubulin-mediated endocytosis in the renal proximal tubule (Figure 2). However, in extrarenal tissues, uptake of 25(OH)D most likely depends on its freely circulating form, rather than the megalin-dependent uptake of the 25(OH)D-DBP complex. Renal hydroxylation of 25(OH)D to calcitriol by the enzyme CYP27B1 is usually tightly regulated by PTH and the phosphaturic hormone fibroblast growth factor-23 (FGF23) in order to maintain plasma calcium (Ca) and inorganic phosphate (P_i_, P if phosphorus is meant) levels within a relatively narrow physiological range (see below). After release into the bloodstream, 85–88% of calcitriol binds to DBP, and 12–15% to albumin. Only about 0.4% of calcitriol is present in free form in the blood [13], and only this freely circulating calcitriol is taken up into the target cells by vitamin D receptors. They are present in almost all human tissues. Since calcitriol is a steroid hormone, it exerts genomic effects via cytosolic receptors and rapid, non-genomic effects in the cell via membrane-bound receptors. Cytosolic ligand-bound VDR heterodimerizes with retinoid-X-receptor (RXR), and this complex is translocated into the cell nucleus, where it modulates the expression of 200–800 genes [13]. The inactivation of both 25(OH)D and calcitriol is initiated by the renal enzyme CYP24A1 (24-hydroxylase) to 24,25-dihydroxyvitamin D(24,25(OH)_2_D) and 1,24,25-trihydroxyvitamin D (1,24,25(OH)_3_D), respectively (Figure 1). Through several further intermediates, CYP24A1 ultimately metabolizes 1,24,25-trihydroxyvitamin D to the biliary excretion product calcitroic acid. Additionally, 25(OH)D and calcitriol are degraded by hepatic CYP3A4. This C23-oxidative pathway catalyzes 25(OH)D_3_ to 25(OH)D_3_-26,23-lactone and calcitriol to 1,25(OH)_2_D_3_-26,23-lactone, two further degradation products, which are secreted in the bile [14].

### 2.2. Regulation of Calcitriol Synthesis

In healthy adults, circulating calcitriol concentrations are tightly regulated by PTH- and FGF23-mediated processes in response to changes in serum ionized calcium (Ca) and Pi levels (Figure 2). The reference range of calcitriol is considered to be 38–134 pmol/L (divide by 2.4 to convert pmol/L to pg/mL) [15]. Serum/plasma calcitriol can be determined using different methods such as radio-immunoassay, enzyme-linked immunoassay, chemiluminescence immunoassay, and liquid chromatography tandem mass spectrometry. PTH and FGF23 exhibit calciotropic effects (PTH) and/or have phosphaturic properties (PTH, FGF23) [16]. In the case of low serum ionized calcium, PTH is secreted by the parathyroid glands by activation of a Ca-sensing receptor (CaSR) [17]. PTH (reference range: 10–60 pg/mL) facilitates hydroxylation of 25(OH)D to calcitriol in the kidneys [13]. At a permissive calcitriol level, PTH also stimulates Ca resorption from bone tissue by activating osteoclasts. Moreover, PTH increases Ca reabsorption in the renal tubules. The effect of PTH on the renal tubules also leads to decreased Pi reabsorption and increased renal P_i_ excretion due to lowering of the sodium/P_i_ cotransporters [17]. PTH and renal 1α-hydroxylation of 25(OH)D is suppressed by high serum Ca levels [16].

As mentioned above, renal CYP27B1 is also suppressed by FGF23. The reference range for the frequently used assays of intact and c-terminal FGF-23 is 10–50 pg/mL and 23–95 RU (research units)/mL, respectively. FGF23 is secreted in mesenchymal cells such as osteocytes, osteoblasts, and odontoblasts, and binds to its cell surface receptor with much higher affinity in the presence of a cofactor, called klotho. Like PTH, FGF23 is stimulated by high serum P_i_ and promotes phosphaturia by reducing renal P_i_ reabsorption to maintain serum P_i_ levels within the normal range [17]. Suppressive effects of FGF23 on CYP27B1 result in reduced circulating calcitriol. In addition, FGF23 activates CYP24A1, resulting in an increased metabolism of 25(OH)D to 24,25(OH)_2_D [14]. The intrinsic activity of renal CYP24A1 is low, but can be increased 20,000-fold [18]. In addition to FGF23, the activity of CYP27B1 is suppressed by its end product calcitriol, while CYP24A1 is suppressed in the presence of PTH, and CYP27B1 is activated by PTH [19]. Thus, CYP24A1 and CYP27B1 are reciprocally expressed to regulate circulating calcitriol [20].

### 2.3. Regulation of Plasma Calcium and Phosphorus

Circulating calcitriol plays a pivotal role in the regulation of Ca and P metabolism by increasing the efficacy of intestinal absorption of Ca and P [1]. Moreover, calcitriol increases renal Ca and P reabsorption, as well as Ca and P resorption from bone. In healthy adults, total serum Ca and ionized serum Ca ranges between 2.17 and 2.51 mmol/L and 1.12–1.32 mmol/L, respectively [20]. An oral Ca bolus of 1000 mg increases postprandial mean ionized serum Ca by only 4–7%, i.e., 0.05 to 0.08 mmol/L [21]. Serum P_i_ is less tightly regulated than Ca. Its physiologic concentration varies between 0.85 mmol/L at a P intake of 700 mg/d and 1.40 mmol/L at an intake of 4000 mg/d [22]. This relationship holds only in adults with adequate renal function. In these individuals, excess absorbed P can be adequately excreted into the urine up to an intake of 4000 mg/d. Since P is so ubiquitous in various foods, near-total starvation is required to produce dietary P deficiency. Inadequate P intake results in hypophosphatemia, leading to anorexia, anemia, muscle weakness, bone pain, rickets and osteomalacia, general debility, confusion, and even death. Excess P intake results in hyperphosphatemia, leading to calcification of non-skeletal tissues [22]. In high-income countries such as the United States, dietary P intake in men and women is 1605 mg/d and 1186 mg/d, respectively, and thus clearly exceeds the recommended intake of 700 mg daily. However, even the 95 percentiles do not exceed the upper tolerable intake level of 4000 mg/d (mean intake males: 2412 mg/d; females: 1693 mg/d) [23]. Therefore, dietary P intake is safe with respect to deficiency and toxicity. In contrast, mean dietary Ca intake in men and women is often below the recommended dietary intake of 1000 mg/d, and secondary hyperparathyroidism often originates from inadequate dietary Ca intake [24]. With respect to calcitriol, the hormone can double the intestinal absorption rate of Ca from 10–15% to 30–40% in apparently healthy individuals, whereas calcitriol increases intestinal absorption rate of P only by 30%, i.e., from 60% to 80% [1]. Ca is primarily (80%) excreted in the feces (sum of unabsorbed and endogenously secreted Ca), whereas P is primarily (70%) excreted in the urine.

## 3. Nutritional Factors Influencing Circulating Calcitriol

### 3.1. Vitamin D and Calcium Supplementation

#### 3.1.1. Rickets and Osteomalacia

Although circulating 25(OH)D is usually 500–1000 times higher than circulating calcitriol, substrate deficiency can lead to low circulating calcitriol concentrations [25]. Classical severe vitamin D deficiency illnesses are nutritional rickets in children and osteomalacia in adults. Both bone diseases are associated with secondary hyperparathyroidism and low serum Ca and P_i_ concentrations [26]. Nutritional rickets can result in muscle weakness, hypotonia, seizures, growth retardation, pneumonia, and death. Circulating 25(OH)D is usually at a level < 25 nmol/L [26], but even with adequate calcium intake the risk of rickets begins already to increase non-linearly at a circulating 25(OH)D level < 50 nmol/L [27], suggesting that circulating calcitriol and thus the calcium absorption rate becomes inadequate. This is consistent with a study in children showing that vitamin D supplementation increased circulating calcitriol when baseline 25(OH)D was <50 nmol/L [11]. Nevertheless, rickets can still occur, even at a circulating 25(OH)D level of 100 nmol/L [27], indicating that in this case it is not primarily the absorption rate but the calcium intake that is deficient. In Europe and North America, a combination of both vitamin D and calcium deficiency may have been responsible for the very high prevalence of rickets during industrialization and urbanization in the 19th and early 20th century. In children with rickets, circulating calcitriol has been reported to be normal, low, or high [28], but systematic investigations indicate higher circulating calcitriol concentrations in rachitic than in non-rachitic children, particularly in patients with deficient calcium intake [29]. In these cases, normal or elevated levels of calcitriol have been assumed to be not sufficiently high to meet the increased Ca requirements associated with the generalized mineralization defect and increased bone turnover. Support for this hypothesis comes from data which show that in rickets calcitriol concentrations rise from mean levels of 73 pmol/L (rickets stage 1), 217 pmol/L (rickets stage 2), and 76 pmol/L (rickets stage 3) to mean levels > 400 pmol/L after vitamin D supplementation with 5000 to 10,000 IU [25]. Intestinal Ca absorption may reach 80% of dietary Ca intake during this phase [26]. The need for high circulating calcitriol in rickets may result in additional substrate deficiency and may further reduce circulating 25(OH)D [28]. Altogether, the results support the assumption that in many cases, both vitamin D and calcium deficiency contribute to the risk of rickets. In patients with nutritional rickets, FGF23 concentrations are within the reference range [30] and Table 1. In rare cases, rickets can also be caused by patients’ intrinsic resistance to vitamin D (see Section 7).

Osteomalacia leads to demineralization of bones, causing them to break more easily. Particularly, the risk of femoral neck and hip fractures is increased. In addition to bone pain, muscle weakness and stiffness are frequent symptoms of osteomalacia. A few studies have reported data on circulating calcitriol in patients with osteomalacia [31,32,33]. In affected patients, baseline circulating calcitriol was low (<41 pmol/L) or undetectable [31,33], and 25(OH)D was below 25 nmol/L [31,32,33]. Treatment with small doses of vitamin D (200–450 IU/d) resulted in a rise in calcitriol to supra-physiological values (500 pmol/L within the first two weeks of treatment) and remained high during the next weeks [33]. Circulating 25(OH)D rose from below 6 nmol/L to 25 nmol/L. Given the increase in circulating calcitriol of about 450 pmol/L, in that study and the supplement-induced increase in circulating 25(OH)D of 19 nmol/L, the conversion rate of 25(OH)D to calcitriol was 1:42, and thus much higher than the conversion rate of 1:500 to 1:1000 in healthy individuals. However, results are in line with aforementioned data [12], indicating that radioactive-labeled vitamin D is converted to calcitriol at a much higher rate in patients with osteomalacia than in healthy individuals. In the aforementioned study [33], 24,25(OH)_2_D was undetectable during the first two weeks of treatment, and its delayed appearance was not related to any other measured variable.

#### 3.1.2. General Population

An MA of randomized controlled trials (RCTs) has summarized data regarding the effect of vitamin D supplementation on circulating calcitriol in apparently healthy adults and different groups of patients with a mean baseline 25(OH)D of 47 nmol/L. In that MA, the mean baseline calcitriol concentrations were 100 pmol/L [34]. Vitamin D supplementation resulted in a mean increase in circulating calcitriol of 12 pmol/L. Although this increase is substantially lower than in patients with rickets or osteomalacia, it is still significant. A subgroup analysis reported small differences regarding vitamin D dose (>1000 IU/d vs. ≤1000 IU/d), mean baseline 25(OH)D (<50 nmol/L vs. ≥50 nmol/L), and kidney function (with or without chronic kidney disease), but these differences did not achieve statistical significance.

Another meta-analysis of RCTs has demonstrated that in apparently healthy individuals with initial 25(OH)D < 50 nmol/L and ≥50 nmol/L, vitamin D supplementation suppresses serum PTH on average by 17 pg/mL and 2 pg/mL, respectively, indicating a threshold of PTH concentrations around 50 nmol/L of 25(OH)D [35]. With respect to FGF23, a meta-analysis reported a significant, but modest incremental effect on FGF23 of a vitamin D dose equivalent ≤ 2000 IU/day, a higher incremental effect of a vitamin D dose equivalent > 2000 IU/day [36], and highest incremental effects by administration of activated vitamin D. The effect was also higher if baseline 25(OH)D was <50 nmol/L instead of ≥50 nmol/L, and this has been at least in part explained by a higher intestinal P absorption until a plateau of P absorption is reached at circulating 25(OH)D ≥ 50 nmol/L.

As mentioned in the paragraph concerning rickets, vitamin D and dietary Ca can replace each other to some extent regarding their effect on human Ca metabolism [37]. A subgroup analysis of the aforementioned meta-analysis of RCTs [34] revealed a significantly smaller increase in circulating calcitriol in RCTs with co-administration of Ca (mean Ca intake: 820 mg/d; range 250–2000 mg/d) compared to vitamin D supplementation alone (5.5 pmol/L vs. 17.3 pmol/L), demonstrating a mean suppressive effect by Ca supplementation of about 12 pmol/L. The data again suggest that an increase in calcitriol occurs as a compensatory consequence of insufficient calcium intake. However, the results also show that this increase after vitamin D supplementation only occurs if the vitamin D status prior to vitamin D supplementation was inadequate. The huge increase in circulating calcitriol in rickets and osteomalacia after vitamin D administration therefore indicates both a severely prior deficient vitamin D status and calcium intake.

#### 3.1.3. Obesity

It has long been known that obesity is related to reduced circulating 25(OH)D and calcitriol concentrations. In a study by Konradsen et al. [38], those with BMI > 39.9 kg/m^2^ had 24% lower 25(OH)D (63 vs. 83 nmol/L) levels and 18% lower calcitriol levels (98 vs. 120 pmol/L) than those with BMI < 25 kg/m^2^. A higher distribution volume and a decreased hepatic CYP2R1 activity have been made responsible for the low circulating 25(OH)D concentration [39,40]. Meta-analyzed data support the assumption of substrate deficiency as a cause of reduced circulating calcitriol concentrations [41,42,43,44,45]. Using a random effect model (I^2^ = 92%; *p* < 0.001), vitamin D supplementation increased circulating calcitriol by 21.9 pmol/L (95% CI: 35.3 to 8.4 pmol/L; *p* < 0.001) (Figure S1A), but publication bias cannot be excluded (Figure S1B). In the subgroups of overweight (four cohorts) and obese (five cohorts) individuals, the effect on circulating calcitriol was 30.1 pmol/L (95% CI: 58.6 to 1.6 pmol/L; *p* < 0.001) and 18.6 pmol/L (95% CI: −5.1 to 42.3 pmol/L; *p* = 0.12). Vitamin D supplementation of the included studies differed substantially and ranged from 400 IU/d [41,42] to 4000 IU/d [43] to 50 µg/d colecalcifediol [45]. In one study [44], a single oral bolus of 300,000 IU vitamin D was administered.

### 3.2. Phosphorus Supplementation

Figure S2A summarizes the results of a meta-analysis of five studies with six cohorts of adults regarding the effect of P supplementation on circulating calcitriol [46,47,48,49,50]. Studies lasted between five days and four weeks. Investigations on acute effects (<2 days) of phosphorus administration were excluded from data analysis. Five cohorts consisted of healthy individuals and one cohort [49] of patients with chronic kidney disease (CKD). The mean supplement dose was 1541 mg/d, ranging from 750 mg/d to 2300 mg/d. P supplementation decreased circulating calcitriol by 15.0 pmol/L (95% CI: −20.2 to −9.9 pmol/L) (Figure S2A) and by 16.9 pmol/L (95% CI: −23.4 to −10.3 pmol/L) if the cohort of CKD patients was excluded. There was no significant heterogeneity between studies (I^2^ = 14%; *p* = 0.33). The magnitude of the P effect on circulating calcitriol was similar to the effect of Ca supplementation, but opposed the direction of vitamin D supplementation. PTH increased only slightly by 4.1 pg/mL (95% CI: 2.9 to 5.3; *p* < 0.001), without evidence of heterogeneity (I^2^ = 0%; *p* = 0.71). In five cohorts with available iFGF23 (47–50), concentrations did not change significantly (mean: 5.6 pg/mL; 95% CI: −0.5 to 11.6 pg/mL; *p* = 0.07), whereas in three cohorts with available c-FGF23 [46,48,50] mean concentrations increased by 11.9 RU/mL (95% CI: 1.1 to 22.6 RU/mL).

### 3.3. Magnesium Intake

Magnesium is a cofactor for CYP27B1. Therefore, it has been assumed that low magnesium status may adversely affect renal calcitriol synthesis and circulating calcitriol [51]. However, a large cross-sectional study could not provide evidence for a significant association between serum magnesium and circulating calcitriol [52]. Likewise, magnesium administration did not influence circulating calcitriol [53,54].

## 4. Mechanical Loading/Unloading and Circulating Calcitriol

### 4.1. Physical Activity

Physical activity is considered preventive against various chronic diseases [55,56]. At the metabolic level, physical activity influences human Ca metabolism, in addition to various other biochemical metabolic pathways, and knowledge of these changes is central for the understanding of the effects on circulating calcitriol. Briefly, acute effects of aerobic exercise are a decrease in ionized Ca and an increase in PTH [57]. Likewise, exercise increases the release of ionized Ca from the sarcoplasmatic reticulum and also its reuptake rate [58], effects which are considered to be beneficial with respect to cardiovascular disease [59]. The Ca concentration in sweat is not altered by physical exercise [60], but high sweating may increase dermal Ca loss substantially [61]. Urinary Ca loss is acutely reduced by an exercise bout [62]. However, substantially higher Ca intakes have been reported in physically active individuals compared to sedentary individuals, resulting in significantly higher urinary Ca excretion [61]. Physical activity is associated with enhanced intestinal Ca absorption rates [61]. Especially weight-bearing exercise is also related to enhanced bone mineral content and strength [63,64].

With respect to circulating calcitriol, exercise-trained young men have 30% higher levels compared with age-matched sedentary controls [61]. Some studies [65,66,67,68] have systematically investigated the effect of physical activity on circulating calcitriol. The studies lasted one month [65,67], two months [68], and three months [66], and were performed in young males [68], young females [65], male smokers [67], and middle-aged adults [66]. Two studies [65,66] consisted of different exercise intensities, and one study [67] investigated the effect of aerobic exercise with and without vitamin D supplementation. Summary results are depicted in Figure S3A. Data demonstrate an exercise-induced increase in circulating calcitriol of 9.2 pmol/L (95% CI: 6.3–12.0 pmol/L). There was no significant heterogeneity between study results (I^2^ = 26%; *p* = 0.22). In contrast to physical activity, one year of hypokinesia (diminished movement) resulted in trained and untrained individuals in a marked mean circulating calcitriol decrease of 56% and 35%, respectively, whereas calcitriol concentrations remained constant during that time in trained and untrained controls. Hypokinesia was paralleled by significant increases in serum and urinary Ca and P_i_ [69]. Since dietary Ca and P intake did not change during the hypokinetic period, it can reliably be assumed that the changes in Ca and P_i_ metabolism were related to reduced skeletal loading. Although exercise-induced changes in ionized Ca and PTH can be attenuated by Ca supplementation [70,71], Ca supplements did not prevent the negative Ca balance in hypokinetic individuals [72].

### 4.2. Bedrest

The removal of regular weight-bearing activity generates a skeletal adaptive response in humans, resulting in a loss of bone mineral. Bedrest is a human model of disuse osteoporosis [73]. Bedrest results in a decrease in PTH, an increase in urinary and fecal Ca loss, negative Ca balance, an increased risk of stone formation, and a loss of bone mineral density [74]. In seven studies with eight cohorts of healthy individuals [75,76,77,78,79,80,81], lasting 10 to 112 days, mean circulating calcitriol decreased by 24.5 pmol/L (95% CI: −31.1 to −17.9 pmol/L) during bedrest (Figure S4A). There was no statistically significant heterogeneity between studies (I^2^ = 0%; *p* = 0.54). Data in experimental animals show that the decrease in circulating calcitriol after skeletal unloading is only transient. Bone Ca stabilized at approximately 70% of control values [82]. Even chronic calcitriol infusion did not prevent the bone changes induced by acute unloading, indicating that there is no direct involvement of calcitriol in the bone changes induced by skeletal unloading. Likewise, antioxidants [83] and alkaline supplements [84] did not affect bone turnover markers during bedrest, and vitamin D supplementation had minor effects on PTH and bone turnover markers in vitamin D-deficient bedridden older patients [85].

In the aforementioned bedrest studies, PTH decreased by 5.5 pg/mL [95% CI: 2.8–8.2 pg/mL; *p* < 0.001) and in a small dry immersion bedrest study, FGF23 did not change significantly, despite an increase in bone turnover markers [86]. In immobilized experimental rats, calcitriol decreased significantly, paralleled by an increase in the mRNA levels of renal CYP24A1 and a decrease in renal CYP27B1 [87].

## 5. Pregnancy

Pregnancy is associated with a markedly increased Ca demand resulting from the fetal Ca need, skeletal Ca deposition for lactation, and elevated renal Ca loss due to increased glomerular filtration rate (GFR) [88,89]. Increased renal synthesis of calcitriol acts to meet the Ca demands of gestation [88]. Mean circulating calcitriol is at least twice as high in pregnant women at term than in nonpregnant women (197 vs. 91 pmol/L) [90]. Circulating calcitriol starts to increase in the first trimester. Circulating 25(OH)D decreases during pregnancy [91]. There is evidence that PTH remains unchanged or low during pregnancy, whereas other factors such as prolactin, placental lactogen, and PTH-related peptide are able to upregulate intestinal calcium absorption, even in the absence of calcitriol or VDR [91]. Due to elevated circulating calcitriol, FGF23 may increase during pregnancy, whereas serum Pi remains unaltered [91]. In contrast to uncomplicated pregnancies, significantly reduced ionized serum Ca concentrations [91,92] and reduced urinary Ca excretion have been reported in pregnant women with preeclampsia [93,94,95,96]. Moreover, an MA reported lower circulating 25(OH)D in pregnant women with preeclampsia than without preeclampsia [97]. In addition, meta-analyzed data [95,96,98,99,100] show 32.5 pmol/L lower (95% CI: −13.8 to −51.3 pmol/L; *p* < 0.001) circulating calcitriol concentrations in pregnant women with preeclampsia than in pregnant controls (Figure S5A). There is evidence that the relatively low calcitriol concentrations in pregnant women with preeclampsia are due to enhanced glucocorticoid levels [101]. However, publication bias cannot be excluded (Figure S5B).

## 6. Diseases

### 6.1. Chronic Kidney Disease

Renal impairment has a very profound effect on circulating calcitriol. When renal function declines below a GFR of 90 mL/min/1.70 m^2^, the two phosphaturic hormones PTH and FGF23 increase gradually to preserve normophosphatemia at the cost of increased P excretion. When estimated GFR falls below 30 mL/min/1.73 m^2^ (CKD stages 4 + 5), renal failure no longer adequately guarantees renal P excretion despite increasing values of PTH and FGF23, thus leading to hyperphosphatemia [102,103]. In parallel, mean circulating calcitriol declines linearly from 100 pmol/L at CKD stage 1 (GFR > 90 mL/min/1.73 m^2^), to 84–60 pmol/L at CKD stage 2 (GFR: 90–60 mL/min/1.73 m^2^), to 60–50 pmol/L at CKD stage 3 (GFR: 60–30 mL/min/1.73 m^2^), and to 36 pmol/L at CKD stage 4 (GFR: 15–30 mL/min/1.73 m^2^) [104]. In addition, the prevalence of patients with hyperparathyroidism increases simultaneously from almost zero to 95% [104]. This is associated with bone mineral disease, which generally becomes apparent in CKD stage 3, when serum P_i_, FGF23, and PTH increase progressively [105]. In CKD stage 5, mean c-FGF23 can rise to >1000 RU/mL [106], while in anuric patients iFGF23 values of 10,000 and more have been reported [107]. Changes in serum Pi and FGF23 levels in CKD patients increase CYP24A1 expression, resulting in decreased 25(OH)D and calcitriol concentrations [108]. As a consequence, intestinal calcium absorption rate decreases, and PTH secretion is stimulated. So, serum Ca may stay normal, decrease, or increase [105]. Although low calcitriol and increased CYP24A1 expression would suggest higher 24,25(OH)_2_D concentrations, there is a surprising gradual decrease in 24,25(OH)_2_D concentrations at CKD stages 3 to 5 [109], indicating reduced 25(OH)D catabolism to 24,25(OH)_2_D at worsened CKD [110]. In patients with CKD and end-stage renal disease, left ventricular hypertrophy, a risk factor for sudden cardiac death and heart failure (HF), is a very common finding, affecting up to 74% of patients [111].

### 6.2. Heart Failure

Heart failure is a condition in which the heart is no longer able to pump oxygen-rich blood to the rest of the body efficiently.

#### 6.2.1. Rickets/Osteomalacia

Several cases of HF in infants with rickets, hypocalcemia, hypophosphatemia, and elevated PTH have been described [112]. Likewise, a case of nutritional osteomalacia in a middle-aged woman with hypocalcemia and HF has been reported [113].

#### 6.2.2. Non-Osteomalacic Adult Population

Adult HF patients without osteomalacia also have impaired vitamin D status, with significantly lower 25(OH)D and calcitriol levels compared to elderly controls [59]. The lowest calcitriol was reported in HF patients with early onset of the disease. Similarly to CKD, serum Ca may be normal, low, or elevated [114]. In community-based individuals, higher serum P_i_ was associated with greater left ventricular mass cross-sectionally, and with an increased risk of HF prospectively [115]. Likewise, early-onset HF patients had significantly higher P_i_ and higher PTH levels, but lower serum Ca levels than controls, despite preserved kidney function [59]. In patients with end-stage HF, hyperparathyroidism and markedly elevated c-terminal FGF23 levels were reported [116], with 98.7% of FGF23 values above the reference range. In addition, 92.2% of patients had suppressed circulating calcitriol levels, indicating that the failing heart is associated with massive derangements in calciotropic/phosphaturic hormones. In studies examining end-stage HF and heart transplant recipients, circulating calcitriol was inversely associated with poor clinical outcome [117,118,119], and FGF23 predicted strongly and inversely circulating calcitriol [120].

## 7. Genetic Disorders

### 7.1. Vitamin D-Dependent Rickets

Genetic disorders have been reported in the biosynthesis, cellular action, and catabolism of calcitriol. Briefly, vitamin D-dependent rickets (VDDR)-1A is a rare autosomal recessive disorder caused by mutations in the CYP27B1 gene. More than 80 different CYP27B1 mutations are known. The defects affect renal calcitriol synthesis, but are also associated with impaired synthesis of calcitriol in extrarenal cells, such as keratinocytes and macrophages. Calcitriol concentrations remain detectable but are in the deficiency range (4–40 pmol/L) [121,122]. VDDR-1B is caused by homozygote mutations in the CYP2R1 gene. Only a handful of mutations with very low 25(OH)D have been described. Circulating calcitriol is normal or low. Heterozygous loss-of-function mutations in the CYP2R1 gene account for vitamin D deficiency and decreased responsiveness of 25(OH)D to vitamin D supplementation to a lesser degree. It has been suggested that unrecognized heterozygosity for complete loss-of-function mutations is likely to be an underestimated cause of low circulating 25(OH)D concentrations [123].

VDDR-2 is caused by end-organ resistance, amongst them VDDR-2A, a rare recessive genetic disorder in the VDR by defects in signal-transducing proteins [124]. The patients have markedly elevated circulating calcitriol concentrations (>300 pmol/L and up to 1000 pmol/L during treatment). VDDR-2B is due to abnormal expression of a hormone response element binding protein that interferes with the normal function of the vitamin D receptor. So far, only one patient has been described [124].

VDDR-3 is due to heterozygote gain-of-function mutations in the CYP3A4 gene, leading to accelerated degradation of vitamin D metabolites such as 25(OH)D and calcitriol [123]. Circulating 25(OH)D is low, and circulating calcitriol is depressed or undetectable. Very few patients with CYP3A4 mutations have been identified. Under physiologic conditions, the relevance of CYP3A4 in inactivating vitamin D metabolites is low. However, compared with its wildtype, the mutant CYP3A4 had a ten-fold increase in activity to inactivate calcitriol and exhibits a two-fold greater activity than CYP24A1. Notably, expression of CYP3A4 can also be induced by different drugs, such as anticonvulsants and many other drugs, leading to accelerated inactivation of vitamin D metabolites. This mechanism may be important in drug-induced osteomalacia [123].

Biochemically, all types of VDDR have hypocalcemia, hypophosphatemia, and hyperparathyroidism. Clinical signs are similar to nutritional rickets and include early-onset rickets, bone pain, muscle weakness, hypotonia, seizures, growth retardation, pneumonia, and death. VDDR-1A is successfully treated with calcitriol, VDDR-1B with calcifediol, and VDDR-2A with intravenous Ca or supraphysiologic oral Ca. Notably, many children with VDDR-2A exhibit spontaneous improvement of rickets around the time of puberty. This indicates that the pubertal increase in estrogens (which are also produced in males by an aromatase that converts androgens to estrogens) mediates a vitamin D-independent action to increase intestinal Ca absorption [123].

### 7.2. CYP24A1 Mutations

In very rare cases, hypercalcemia can be the result of bi-allelic loss-of-function variants in the CYP24A1 gene. Patients with biallelic CYP24A1 mutations present a PTH-independent hypercalcemia highly variable in its severity, but generally mild outside the settings of early infancy and pregnancy [125]. Circulating 25(OH)D and calcitriol are also highly variable, but are frequently at the upper reference range or above [126]. For detecting CYP24A1 dysfunction, the use of the serum 25(OH)D_3_:24,25(OH)_2_D_3_ ratio is considered an accurate parameter. The normal 25(OH)D_3_:24,25(OH)_2_D_3_ ratio is below 25, whereas ratios above 80 are indicative of CYP24A1 mutations [125]. In a subset of patients diagnosed with idiopathic hypercalcemia and hypercalciuria, monoallelic mutations and polymorphisms affecting CYP24A1 activity have been identified, but the clinical picture is less severe than in bi-allelic loss-of-function variants. This disorder is characterized by hypercalciuria and nephrocalcinosis [126]. Among these patients with reduced CYP24A1 activity, the 25(OH)D_3_:24,25(OH)_2_D_3_ shifted to >25 [19]. The prevalence of CYP24A1 mutations in the general population is unknown, but may contribute to hypercalcemia, a condition with an estimated prevalence of 1/500 patients in the outpatient setting [126].

In experimental mice, the absence of CYP24A1 causes 50% postnatal mortality due to severe hypercalcemia, accompanied by markedly elevated calcitriol and undetectable PTH [127]. Compared to CYP24A1+/− fetuses, the null fetuses are hypercalcemic, modestly hypophosphatemic, and have a 3.5-fold increase in calcitriol and a 4-fold increase in FGF23 [127].

## 8. Extrarenal Calcitriol Synthesis and Regulation

In many tissues, particularly components of the immune system and various epithelia, the enzymatic activity of CPY2R1 and CYP27B1 has been detected (Table 2).

Likewise, the enzyme CYP24A1 is inducible in all calcitriol target tissues [20], indicating that locally synthesized calcitriol is largely degraded before it enters the circulation. It had been assumed that without vitamin D, the ability of the cell to respond adequately to pathologic and physiologic signals is impaired, and that circulating concentrations of 25(OH)D become a critical factor in ensuring optimal functioning of the various systems that require calcitriol for intracrine and paracrine vitamin D actions [129]. However, current data suggest that extrarenal calcitriol synthesis is not simply dependent on circulating 25(OH)D concentrations. Synthesis and regulation have been extensively studied in macrophages/monocytes. Although extrarenal CYP27B1 is identical to renal CYP27B1 [130], calcitriol synthesis in macrophages is not regulated by PTH. Its production markedly depends on pathogen-induced activation of immune responses via toll-like receptors (TLRs), resulting in an induction of the cathelicidin antimicrobial peptide gene. In addition, CYP27B1 is activated by cytokines such as IFNgamma, IL-2, IL-15, IL-32, and TNFα, whereas extrarenal CYP27B1 is suppressed by IL-10. In macrophages, CYP27B1 activity is also suppressed by FGF23, and in serum from patients with end-stage renal failure, who have very high FGF23 concentrations, stimulated CYP27B1 expression and calcitriol synthesis are reduced relative to serum from healthy donors [130]. Despite the obvious importance of CYP27B1 in macrophages for adequate immune response, it is noteworthy that extracellular Ca may also play a pivotal role in macrophage activation and the regulation of key pathogen response pathways, while the role of CYP27B1 in this process is still unclear [130].

In contrast to macrophages, PTH is a stimulator of CYP27B1 in other extrarenal tissues such as keratinocytes, cultured human umbilical vein endothelial cells, and vascular smooth muscle cells [130,131]. However, similar to macrophages, CYP27B1 is stimulated by the TLR4 ligand LPS and TNFα in human umbilical vein endothelial cells and suppressed by FGF23 in cardiomyocytes, keratinocytes, cardiomyocytes, and vascular smooth muscle cells [130,131,132].

It is noteworthy that some patients with a granulomatous disease develop hypercalcemia/hypercalciuria due to elevated extrarenal calcitriol synthesis. The first reports were related to patients with sarcoidosis, but now more than 25 different diseases have been described in which increased circulating calcitriol is most likely of extrarenal origin. The diseases include inflammatory conditions, pathogen-mediated infections, and neoplasms. Affected patients have a frankly high or inappropriately elevated circulating calcitriol concentration, although their serum PTH level is suppressed and their serum Pi concentration is relatively elevated [130]. Their circulating calcitriol concentration is very sensitive to an increase in available substrate [130]. The mechanisms for unregulated calcitriol synthesis in these diseases are still unclear and may be related to dysregulated CYP24A1 activity. Glucocorticoids, chloroquine, and hydroxychloroquine can be used to effectively lower elevated circulating calcitriol. These drugs appear to have a preferential effect on extrarenal CYP27B1.

## 9. Clinical Implications

The results summarized in this article have several major clinical implications. First, as expected, substrate availability can have a substantial impact on circulating calcitriol, whereas in healthy individuals, the effects of physiologic Ca and P doses on circulating calcitriol are small. Second, vitamin D plus Ca supplements can only improve bone health in case of inadequate Ca supply, i.e., (sub)clinical osteomalacia, but cannot prevent osteoporosis due to mechanical unloading. Third, Ca and P metabolism differ in infants with HF from adults with HF, whereas adult HF has several similarities with CKD regarding dysregulated calciotropic and phosphaturic hormones. Finally, basic research indicates that in immune cells, calcitriol synthesis is largely independent of circulating 25(OH)D, a finding that challenges the use of vitamin D supplements to prevent infections.

### 9.1. Vitamin D Supplementation, Rickets/Osteomalacia and Bone Diseases

Rickets prevention by vitamin D supplementation is widely used in different countries, at least in Europe [133]. In recent years, it also became clear that Ca and vitamin D can replace each other, to some extent, in their preventive effects on bone [27,134]. The successful treatment of VDDR types 1A and 2A with calcitriol and Ca administration, respectively, supports the pivotal role of adequate Ca availability for the prevention of rickets (either by calcitriol-mediated optimal Ca absorption rates or supraphysiologic Ca doses in the case of absence of VDR-mediated vitamin D action).

The prevalence of osteomalacia is unclear, but it has been seen on bone biopsy in about 4–5% of general medical and geriatric patients who had not suffered a fracture [4]. A comprehensive MA of RCTs [135] provided no statistically significant evidence for a reduction in total fractures by vitamin D with or without Ca supplementation. Likewise, vitamin D supplementation alone did not reduce hip fracture, but vitamin D co-administered with Ca reduced hip fracture significantly in institutionalized individuals, indicating that in residents of institutions, vitamin D status or calcium intake or both was deficient. It is noteworthy that data are sparse regarding the prevalence and management of osteomalacia. This is due to diagnostic limitations, since for proper diagnosis of osteomalacia, bone biopsy is necessary [136]. This has led to the integration of osteomalacia management within osteoporosis management in adults. However, osteoporosis is a disease primarily occurring due to sarcopenia-induced low mechanical forces [137], whereas nutritional osteomalacia is the result of deficient Ca supply due to vitamin D and/or dietary Ca deficiency. Presently, the possibility cannot be excluded that the reported beneficial effects on hip fractures with vitamin D and Ca supplementation were related to undiagnosed osteomalacia rather than to osteoporosis. In line with this assumption, an MA of RCTs [138] estimated that the effectiveness of vitamin D supplementation in reducing hip fractures is highest in residents in institutions, a group with frequently low circulating 25(OH)D, low dietary Ca, and high PTH concentrations [139]. Data on circulating calcitriol during bedrest and physical inactivity are in line with the assumption that in these cases, the decrease is a consequence of Ca and P release from bone, as the human body merely aims to adapt to a new state by reducing bone mass due to the lower mechanical forces. These individuals require mobilization and weight-bearing exercise rather than nutritional interventions as therapies of choice.

### 9.2. Diabetes Mellitus

Although type 2 diabetes mellitus (T2DM) is not a vitamin D-dependent disease, there is evidence that insulin sensitivity and ß-cell function can be improved in patients with newly diagnosed T2DM by daily vitamin D supplementation of 5000 IU [140]. Both effects seem to be based on VDR-mediated calcitriol action [141,142]. T2DM is usually associated with obesity. In trials testing moderate to high doses of vitamin D supplementation (≥1000 IU/day) among participants with prediabetes, the relative risk of T2DM for vitamin D compared with placebo was 12% lower [143]. Results are in line with data of the present MA in obese individuals, demonstrating an increase in circulating calcitriol by vitamin D supplementation. The subgroup analysis of that MA does not exclude the possibility that patients with higher BMI need higher doses of vitamin D than patients with lower BMI to increase circulating calcitriol. This is also in line with systematic data indicating that after vitamin D supplementation, high body weight leads to a lower increment in circulating 25(OH)D than low body weight [38].

### 9.3. Preeclampsia

Different MAs have demonstrated that both Ca and vitamin D supplements are able to reduce the risk of preeclampsia [144,145,146,147]. The preventive mechanism of Ca and vitamin D is unclear at present, but may be due to rectifying a formerly existing low dietary Ca intake, low ionized serum Ca, relatively low circulating calcitriol, and high intracellular Ca concentrations in preeclamptic pregnant women [147], which may increase the pathophysiologic alterations in this situation.

### 9.4. Chronic Kidney Disease Treatment

The prevalence of deficient 25(OH)D (<25 nmol/L) and low circulating calcitriol is high in CKD [105]. This may contribute to the development of CKD-associated bone disease. Reduced 25(OH)D and calcitriol concentrations in CKD are most likely primarily due to elevated concentrations of the phosphaturic hormone FGF23. Therefore, restricting intestinal P uptake seems to be the best strategy to prevent hyperphosphatemia, hypocalcemia, high FGF23, hyperparathyroidism, and suppressed calcitriol. Although positive results on circulating calcitriol have been reported by P restriction in children with CKD [148], P restriction is not an easy task. Dietary P intake in adults is usually higher than the recommended 700 mg/d [22], and P-rich foods such as cereals, legumes, nuts, dairy products, meat, fish, and eggs are also rich in protein and contribute to a healthy diet. P_i_ binders such as magnesium carbonate are also used to restrict intestinal P uptake, but dialysis patients, for instance, experience a 300–500 mg/day surplus if consuming 900–1500 mg/day of P, while P_i_ binders have a binding capacity of only 250 mg/day. The excess P, if absorbed, can contribute to hyperphosphatemia [149]. Therefore, dual binder therapy has been proposed [150]. Although in CKD administration of vitamin D or active vitamin D may increase circulating calcitriol [34,151] and may thus reduce hyperparathyroidism, this can also increase intestinal P absorption rate [150] and FGF23 [152]. Vitamin D recommendations should therefore follow those of the general population [800–1000 IU], dietary Ca should not substantially exceed the recommended daily intake of 1000 mg, and active vitamin D (in case of CKD stage 4 and 5) should be taken with caution to avoid a further increase in FGF23. If hyperphosphatemia persists, the relative risk of soft-tissue calcifications and cardiovascular disease, as well as all-cause mortality, is increased [105]. Altogether, therapy of CKD is complex, and available evidence suggests that serum P_i_ should be maintained within the reference range, if necessary, by dietary P restriction and use of P_i_ binders [149].

### 9.5. Heart Failure Treatment

Since symptoms of rickets include muscle weakness, it is understandable that some rachitic infants with dilated cardiomyopathy, a form of HF, can be successfully treated with vitamin D and Ca supplements [112]. In these children, hypocalcemia is considered to be an important cause of HF. Although adult HF patients also have lower serum Ca than healthy elderly adults [59], the disease seems to differ from HF in infants with rickets since, in contrast to nutritional rickets in children, serum P_i_ is slightly and FGF23 markedly elevated in adult HF [59,153]. The effect of vitamin D supplementation on left ventricular ejection fraction, an indicator of cardiac function, is inconsistent in HF, since one MA demonstrated an increase in left ventricular ejection fraction [154], whereas another MA reported an increase only in RCTs without co-administration of Ca [155]. In the VITAL trial, vitamin D supplementation did not significantly reduce the incidence of first HF hospitalization [156]. Generally, caution is necessary in supplementing HF patients and other CVD patients with vitamin D, especially if moderately high doses (>4000 IU), which were most effective in enhancing left ventricular ejection fraction [154], are administered. Such doses may also increase the risk of hypercalcemia [157], high serum P_i_ [158], and elevated FGF 23, a situation associated with a high risk of CVD [105]. Genetically predicted lifelong higher concentrations of serum Ca already indicate shortened life expectancy and increased CVD risk [159]. Thus, similar to CKD, the therapeutic window to enhance circulating calcitriol may be narrow in HF.

### 9.6. Acute Respiratory Tract Infection

An aggregated study-level MA [160], based on 40 studies and >60,000 study participants, reported that vitamin D supplementation versus placebo reduced the risk of acute respiratory tract infection only non-significantly (odds ratio 0.94 [95% CI 0.88–1.00]). In pre-specified subgroup analysis, there was no effect modification by age, baseline 25(OH)D status, dosing frequency, or dose size. Data are in line with basic research indicating that in immune cells, calcitriol synthesis is usually largely independent of circulating 25(OH)D. With respect to COVID-19 infection, it has been concluded that evidence for the effectiveness of vitamin D supplementation for the treatment of COVID-19 is very uncertain [161]. In addition, several limitations have been outlined regarding an MA that concluded beneficial vitamin D effects on COVID-19-related outcomes [162]. Moreover, it was stated that strong publication bias affected small RCTs regarding COVID-19 and acute respiratory tract infection [163].

## 10. Conclusions

Calcitriol is an important steroid hormone, and this article gives an overview of various factors influencing circulating calcitriol. However, it has to be acknowledged that only a brief summary could be given and that other factors, such as aging, sex hormones, and glucocorticoids, may also influence circulating calcitriol. In addition, some presented MAs are based on a few studies only, and the effect size of these MAs should therefore be interpreted with caution. Likewise, this article aimed to focus on human data, while the extensive literature regarding vitamin D metabolism and its regulation in experimental animals has only been partly taken into account. Also, only a few diseases were considered, although vitamin D actions are discussed in connection with many other illnesses. Despite these restrictions, some conclusions can be drawn from this overview for clinical implications and future research.

There is mounting evidence that in healthy individuals, changes in serum Ca and P_i_ are the principal regulators of circulating calcitriol. Reduced circulating calcitriol does not necessarily indicate inadequacy, but may sometimes be an adaptation to high Ca release from bone or impaired renal P excretion. Low Ca availability due to deficient circulating 25(OH)D is usually best mirrored by secondary hyperparathyroidism. Data indicate that adequate substrate availability, i.e., adequate vitamin D (plus Ca), not only prevents rickets and osteomalacia but also reduces the risk of T2DM and preeclampsia. High serum P_i_, which leads to low 25(OH)D and calcitriol, but also to increased FGF23 concentrations, is a particular problem in certain diseases such as CKD and HF. Personalized approaches to preventing or treating low vitamin D metabolite concentrations are therefore necessary. This means that (i) vitamin D supplement use is required in individuals with insufficient or deficient vitamin D status, (ii) physical activity should be recommended in case of bedrest/immobilization, and (iii) P restriction is needed in patients with hyperphosphatemia and/or high FGF23 levels.

Since circulating calcitriol is regulated by Ca and P_i_, but can influence several hundred genes in the cell, the development of hypocalcemic vitamin D analogs is an interesting area of research, particularly with respect to the anticancer properties of calcitriol [164]. Regarding extrarenal calcitriol synthesis, work is underway to inject CYP27B1-overexpressed dendritic cells or macrophages in vivo. They then release high concentrations of calcitriol into peripheral lymphoid tissue to stimulate CD4+ Tregs or to enhance the differentiation of stem cells. This could be of therapeutic use, for example, in the suppression of autoimmune diseases [165]. Future research should also elucidate the etiology of elevated serum P_i_ and FGF23 in HF patients. In addition, research should focus on vitamin D doses necessary for preventing preeclampsia, T2DM, and possibly other diseases with insufficient calcitriol synthesis. Likewise, the effects of changes in serum Ca and P_i_ due to immobilization, estrogen deficiency (anorexia nervosa, menopause), and physical inactivity on VDR-mediated cellular calcitriol actions and illnesses such as malignancies and autoimmune diseases should be investigated.

## Figures and Tables

**Table 1:** Effects of nutrients, mechanical loading/unloading, genetic disorders, and specific diseases on serum concentrations of vitamin D metabolites and parameters of calcium and phosphate pathways ^1^.

	25(OH)D	24,25(OH)_2_D	Calcitriol	Serum Calcium	Serum Phosphate	PTH	FGF23
Nutritional rickets/osteomalacia	↓↓↓	↓↓↓	varies	N, ↓	N, ↓	↑↑↑	N, ↓
Vitamin D insufficiency	↓	↓	varies	N	N	N, ↑	N
Calcium supplement	N	N	↓	N	N	N, ↓	N
Dietary phosphate load	N	N	↓	N	N	N, ↑	N, ↑
Hypokinesia	↓	↓	↓ to ↓↓	N	N	N	N
Bedrest	↓ to ↓↓	?	↓↓	N	N	N, ↓	N
Physical activity	N to ↑	N to ↑	↑	N	N	N	N
Pregnancy	↓	↓	↑↑	↓	N	N, ↓	↑
Chronic kidney disease stages 1–3	↓	↓	↓	N	N	↑	↑
Chronic kidney disease stages 4–5	↓ to ↓↓	↓ to ↓↓	↓↓	N, ↓	↑	↑↑	↑↑
End-stage renal disease/hemodialysis	↓ to ↓↓	↓ to ↓↓	↓↓↓	N, ↓	↑↑	↑↑↑	↑↑↑
End-stage heart failure	↓ to ↓↓	?	↓↓ to ↓↓↓	N, ↓	↑	↑↑↑	↑↑↑
Vitamin D-dependent rickets type 1A	↓ to ↓↓	N	↓↓	↓ to ↓↓	N, ↓	↑↑↑	N, ↓
Vitamin D-dependent rickets type 1B	N	↓	varies	↓ to ↓↓	N, ↓	↑↑↑	N
Vitamin D-dependent rickets type 2A	↓↓	?	↑↑	↓ to ↓↓	N, ↓	↑↑↑	N, ↓
Vitamin D-dependent rickets type 2B	N	?	↑↑	↓ to ↓↓	N, ↓	↑↑↑	N
Vitamin D-dependent rickets type 3	N	N?	↓ to ↓↓↓	↓ to ↓↓	↓	↑↑↑	?
CYP24A1 mutations	↓↓	↓ to ↓↓↓	↑ to ↑↑↑	↑ to ↑↑	N, ↓	N	↑↑
Granulomatous diseases	N	?	↑ to ↑↑↑	↑ to ↑↑	↑	↓	?

Abbreviations: 25(OH)D, 25-hydroxyvitamin D; 24,25(OH)_2_D, 24,25-dihydroxyvitamin D; PTH, parathyroid hormone; FGF, fibroblast growth factor; CYP, cytochrome P; N, normal; ↓ suppressed; ↓↓ or ↓↓↓, markedly suppressed; ↑, increased; ↑↑ or ↑↑↑, markedly increased; ?, unknown; ^1^ results regarding nutritional rickets, vitamin D-dependent rickets, and CYP24A1 mutations are based on data by ref. [30], other results are based on data in the text.

**Table 2:** Tissues with CYP2R1 (25-hydroxylase) and CYP27B1 (1α-hydroxylase) activity [40,128].

CYP2R1 Activity	CYP27B1 Activity
-	Kidney
Thyroid gland	Thyroid gland
Pancreas	Pancreas
Bone marrow	Bone marrow
Prostate	Appendix
Retina	Retina
Pituitary gland	Adrenal gland
Thymus	Thymus
Lymph nodes	Lymph nodes
Liver	Liver
Skin	Skin
Testes	Testes
Fat tissue	Fat tissue
Placenta, vagina, uterus	Cardiomyocytes, vascular smooth muscle cells, and vascular endothelial cells

## Supplementary Materials

The following supporting information can be downloaded at: https://www.mdpi.com/article/10.3390/ijms26125570/s1.

## Abbreviations

The following abbreviations are used in this manuscript:

25(OH)D	25-Hydroxyvitamin D
24,25(OH)2D	24,25-Dihydroxyvitamin D
BMI	Body mass index
Ca	Calcium
CaSR	Calcium-sensing receptor
CKD	Chronic kidney disease
CYP	Cytochrome P
DBP	Vitamin D binding protein
GFR	Glomerular filtration rate
IFN	Interferon
IL	Interleukin
IU	International unit
FGF	Fibroblast growth factor
HF	Herat failure
LPS	Lipopolysaccharide
P	Phosphorus
Pi	Inorganic phosphate
PTH	Parathyroid hormone
RCT	Randomized controlled trial
TNF	Tumor necrosis factor
TLR	Toll-like receptor
VDR	Vitamin D receptor
VDDR	Vitamin D-dependent rickets
