# Supplementary material for: Regulation of Renal and Extrarenal Calcitriol Synthesis and Its Clinical Implications
Source: Int J Mol Sci. 2025 Jun 11;26(12):5570. doi: 10.3390/ijms26125570 (PMC12192668; doi:10.3390/ijms26125570)
Supplement: Supplementary file 1 [file ijms-26-05570-s001.zip › ijms-3648344-supplementary.pdf]

Supplemental Material

Regulation of renal and extrarenal calcitriol synthesis and its clinical implications

Zittermann A

		Page
**Figure S1A:**	Meta-analysis regarding the effect of obesity on circulating calcitriol	2
**Figure S1B:**	Funnel plot regarding the effect of obesity on circulating calcitriol	2
**Figure S2A:**	Meta-analysis regarding the effect of phosphorus supplementation on circulating calcitriol	3
**Figure S2B:**	Funnel plot regarding the effect of phosphorus supplementation on circulating calcitriol	3
**Figure S3A:**	Meta-analysis regarding the effect of exercise on circulating calcitriol	4
**Figure S3B:**	Funnel plot regarding the effect of exercise on circulating calcitriol	4
**Figure S4A:**	Meta-analysis regarding the effect of bedrest on circulating calcitriol	5
**Figure S4B:**	Funnel plot regarding the effect of bedrest on circulating calcitriol	5
**Figure S5A:**	Meta-analysis regarding the effect of preeclampsia on circulating calcitriol	6
**Figure S5B:**	Funnel plot regarding the effect of preeclampsia on circulating calcitriol	6

Figure S1A: Meta-analysis regarding the effect of obesity on circulating calcitriol

	Baseline			InStudy				Mean Difference
**Study or Subgroup**	**Mean**	**SD**	**Total**	**Mean**	**SD**	**Total**	**Weight**	**IV, Random, 95% CI**
Bhagatwala 2015, a	97	28	17	90	20	17	8.3%	7.00 [-9.36, 23.36]
Bhagatwala 2015, b	101	20	18	85	22	18	8.6%	16.00 [2.26, 29.74]
Bhagatwala 2015, c	102	28	18	79	10	18	8.6%	23.00 [9.26, 36.74]
Canozzi 2016, a	108	46	21	77	33	21	7.2%	31.00 [6.79, 55.21]
Canozzi 2016, b	88	39	14	68	28	14	7.1%	20.00 [-5.15, 45.15]
DAS 2024, a	175	61	267	113	32	267	9.1%	62.00 [53.74, 70.26]
DAS 2024, b	181	64	473	130	43	473	9.2%	51.00 [44.05, 57.95]
Rajakumar 2008	122	30	21	120	53	21	7.0%	2.00 [-24.05, 28.05]
Wood 2014, a	151	50	139	137	43	139	8.9%	14.00 [3.04, 24.96]
Wood 2014, b	150	50	139	137	43	139	8.9%	13.00 [2.04, 23.96]
Wood 2014, c	146	42	53	136	37	53	8.5%	10.00 [-5.07, 25.07]
Wood 2014, d	143	42	53	136	37	53	8.5%	7.00 [-8.07, 22.07]
**Total (95% CI)**			**1233**			**1233**	**100.0%**	**21.97 [8.54, 35.40]**

Heterogeneity: Tau² = 495.88; Chi² = 141.80, df = 11 (P < 0.00001); I² = 92%
Test for overall effect: Z = 3.21 (P = 0.001)

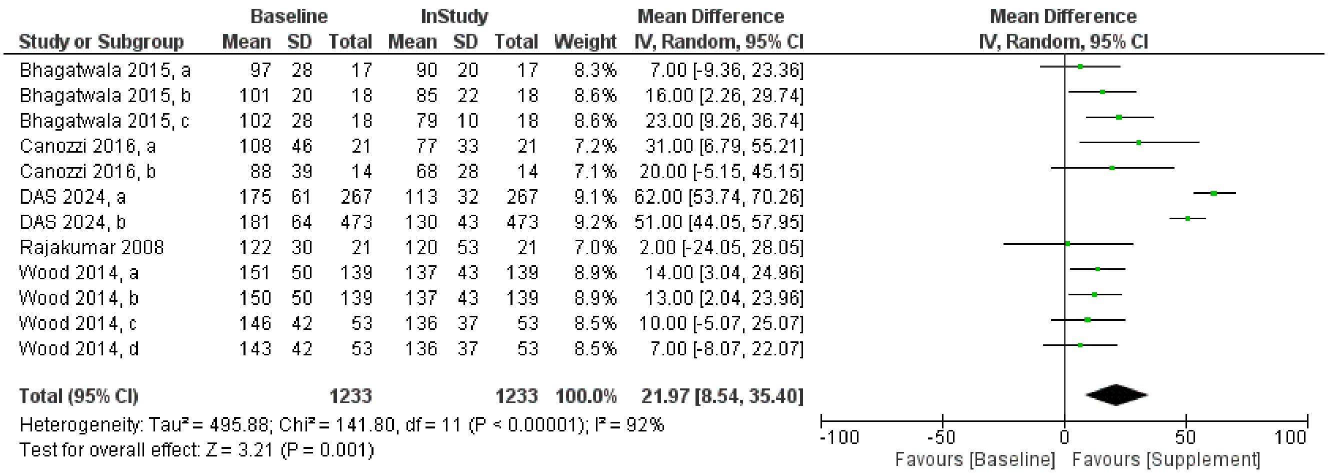

Figure legend: The x-axis indicates the effect on circulating calcitriol in (pmol/l). Negative values indicate a decrease, positive values an increase in circulating calcitriol. Horizontal lines indicate 95% confidence intervals of individual studies

Figure S1B: Funnel plot regarding the effect of obesity on circulating calcitriol

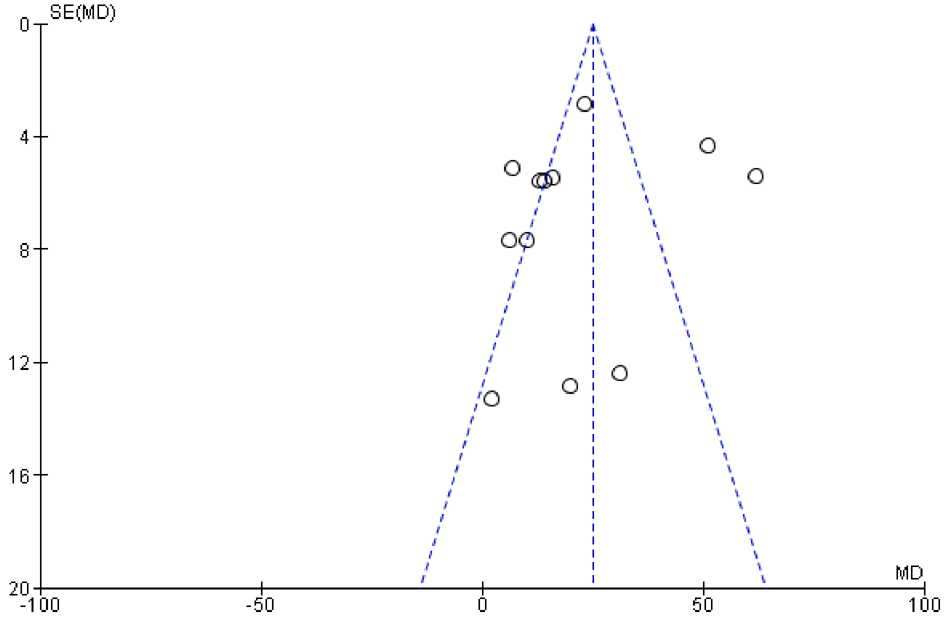

Figure legend: MD indicates mean difference of vitamin D supplementation vs. control in individual trials; each circle displays the result of an individual study; a value < 0 notifies a decrease in circulating calcitriol and a value > 0 notifies an increase in circulating calcitriol by vitamin D supplementation; SE (MD) denotes the standard error of the mean difference. Risk of publication bias cannot be ruled out if circles are lying outside the dotted lines.

Figure S2A: Meta-analysis regarding the effect of phosphorus supplementation on circulating calcitriol

Study or Subgroup	Phosphorus supplemented Mean	SD	Total	Control Mean	SD	Total	Weight	Mean Difference IV, Fixed, 95% CI
Antoniucci 2006	75	26	13	104	42	13	3.7%	-29.00 [-55.85, -2.15]
Burnett 2006	118	53	33	149	70	33	2.9%	-31.00 [-60.96, -1.04]
Ferrari, 2005	85	15	29	95	20	29	31.9%	-10.00 [-19.10, -0.90]
Gerber 2024	110	45	10	128	30	10	2.3%	-18.00 [-51.52, 15.52]
Sigrist 2013,a	88	17	12	111	10	12	21.2%	-23.00 [-34.16, -11.84]
Sigrist 2013,b	51	10	18	63	15	18	38.0%	-12.00 [-20.33, -3.67]
**Total (95% CI)**			**115**			**115**	**100.0%**	**-15.01 [-20.15, -9.88]**

Heterogeneity: Chi² = 5.80, df = 5 (P = 0.33); I² = 14%
Test for overall effect: Z = 5.73 (P < 0.00001)

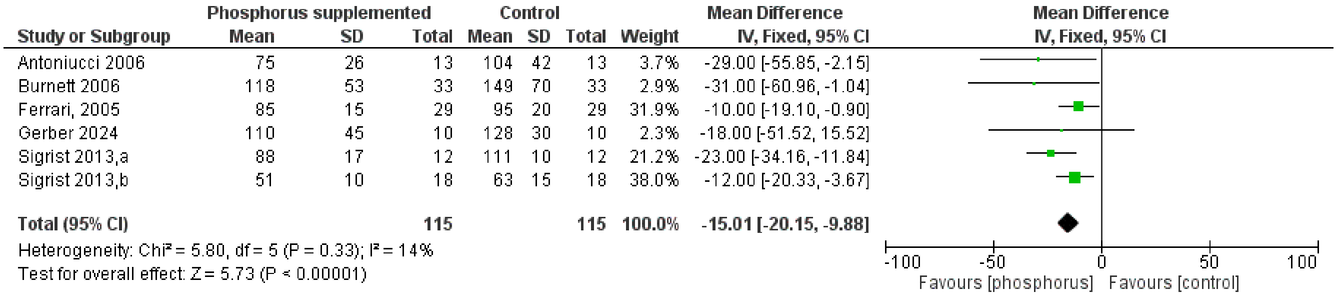

Figure legend: The x-axis indicates the effect on circulating calcitriol in (pmol/l). Negative values indicate a decrease, positive values an increase in circulating calcitriol. Horizontal lines indicate 95% confidence intervals of individual studies

Figure S2B: Funnel plot regarding the effect of phosphorus supplementation on circulating calcitriol

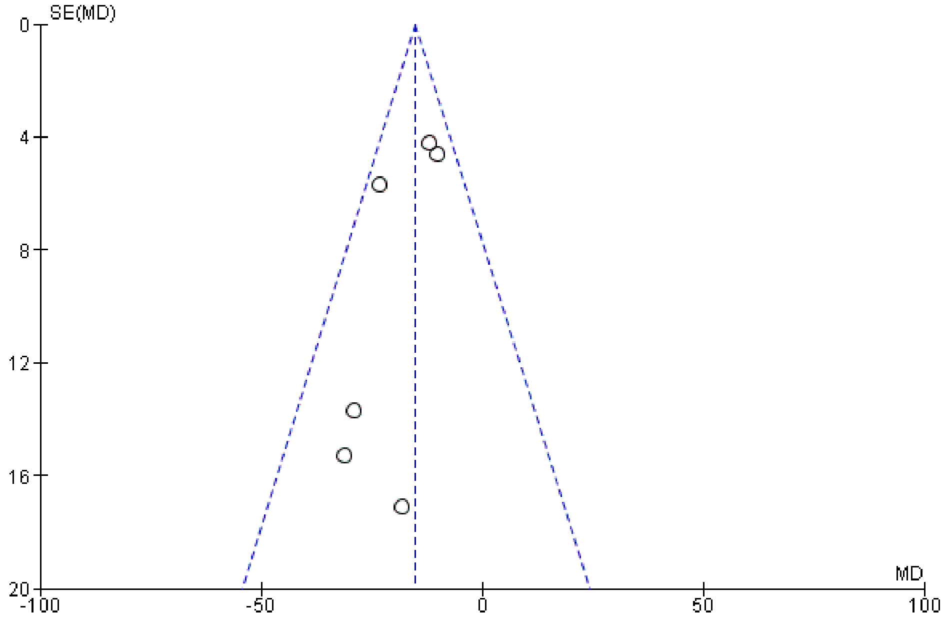

Figure legend: MD indicates mean difference of phosphorus supplementation vs. control in individual trials; each circle displays the result of an individual study; a value < 0 notifies a decrease in circulating calcitriol and a value > 0 notifies an increase in circulating calcitriol by phosphorus supplementation; SE (MD) denotes the standard error of the mean difference. Risk of publication bias cannot be ruled out if circles are lying outside the dotted lines.

Figure S3A: Meta-analysis regarding the effect of exercise on circulating calcitriol

	Experimental			Control				Mean Difference
Study or Subgroup	Mean	SD	Total	Mean	SD	Total	Weight	IV, Fixed, 95% CI
De-la-O 2022, a	116	31	17	102	28	17	2.1%	14.00 [-5.86, 33.86]
De-la-O 2022, b	114	26	16	97	23	16	2.8%	17.00 [-0.01, 34.01]
De-la-O 2022, c	103	43	18	79	39	18	1.1%	24.00 [-2.82, 50.82]
Lu 2022, a	41	8	13	29	6	13	27.8%	12.00 [6.56, 17.44]
Lu 2022, b	36	5	13	29	5	13	55.6%	7.00 [3.16, 10.84]
Nikniaz 2021, a	66	17	10	57	14	10	4.4%	9.00 [-4.65, 22.65]
Nikniaz 2021, b	58	13	10	56	16	10	5.0%	2.00 [-10.78, 14.78]
Tajima 2000	115	77	34	79	24	34	1.1%	36.00 [8.89, 63.11]
**Total (95% CI)**			**131**			**131**	**100.0%**	**9.17 [6.31, 12.04]**

Heterogeneity: $Chi^2 = 9.45$, df = 7 (P = 0.22); $I^2 = 26\%$
Test for overall effect: Z = 6.27 (P < 0.00001)

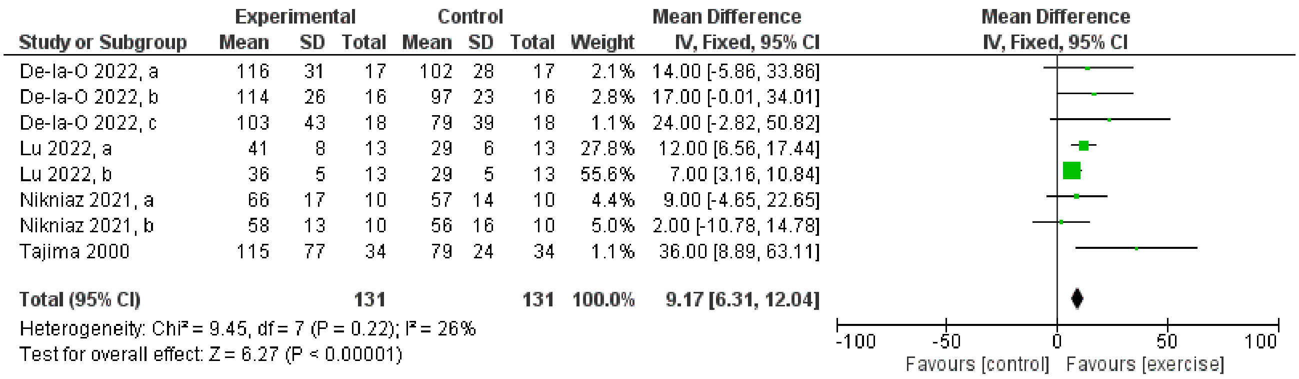

Figure legend: The x-axis indicates the effect on circulating calcitriol in (pmol/l). Negative values indicate a decrease, positive values an increase in circulating calcitriol. Horizontal lines indicate 95% confidence intervals of individual studies

Figure S3B: Funnel plot regarding the effect of exercise on circulating calcitriol

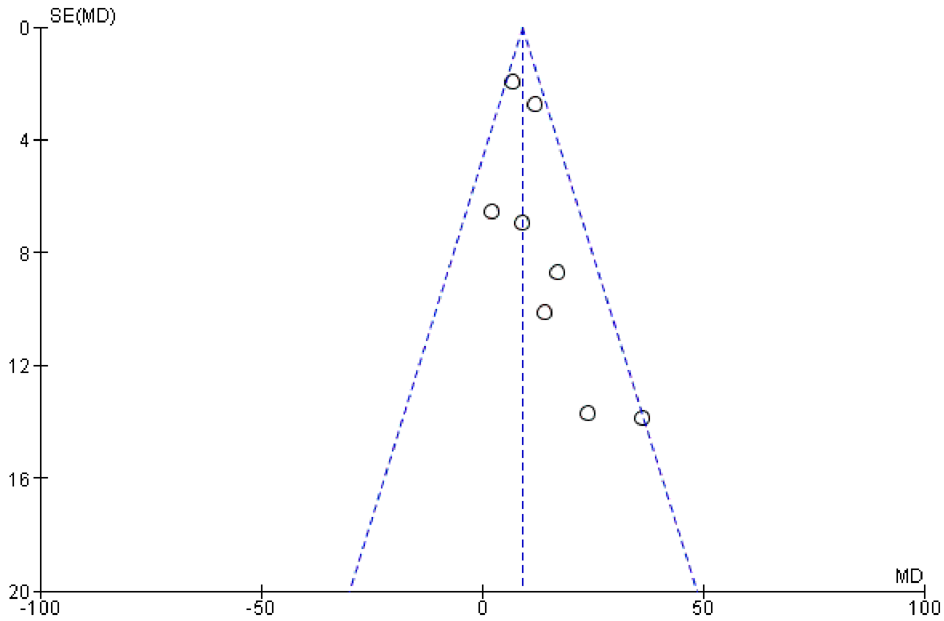

Figure legend: MD indicates mean difference of exercise vs. baseline in individual trials; each circle displays the result of an individual study; a value < 0 notifies a decrease in circulating calcitriol and a value > 0 notifies an increase in circulating calcitriol by exercise; SE (MD) denotes the standard error of the mean difference. Risk of publication bias cannot be ruled out if circles are lying outside the dotted lines.

Figure S4A: Meta-analysis regarding the effect of bedrest on circulating calcitriol

	Experimental			Control				Mean Difference
Study or Subgroup	Mean	SD	Total	Mean	SD	Total	Weight	IV, Fixed, 95% CI
McGrath 2022, a	86	14	8	122	24	8	11.7%	-36.00 [-55.25, -16.75]
McGrath 2022, b	79	36	11	127	41	11	4.2%	-48.00 [-80.24, -15.76]
Morgan 2012	71	13	12	93	18	12	27.6%	-22.00 [-34.56, -9.44]
Scheld 2001	56	23	6	72	22	6	6.7%	-16.00 [-41.47, 9.47]
Smith 2009	163	89	15	147	74	15	1.3%	16.00 [-42.57, 74.57]
van der Wiel 1991	79	36	14	98	33	14	6.7%	-19.00 [-44.58, 6.58]
Zerwekh 1998	46	11	11	70	14	11	39.3%	-24.00 [-34.52, -13.48]
Zwart 2007	101	32	7	126	46	7	2.5%	-25.00 [-66.51, 16.51]
**Total (95% CI)**			**84**			**84**	**100.0%**	**-24.51 [-31.11, -17.91]**

Heterogeneity: $Chi^2 = 6.01$, $df = 7$ ($P = 0.54$); $I^2 = 0\%$
Test for overall effect: $Z = 7.28$ ($P < 0.00001$)

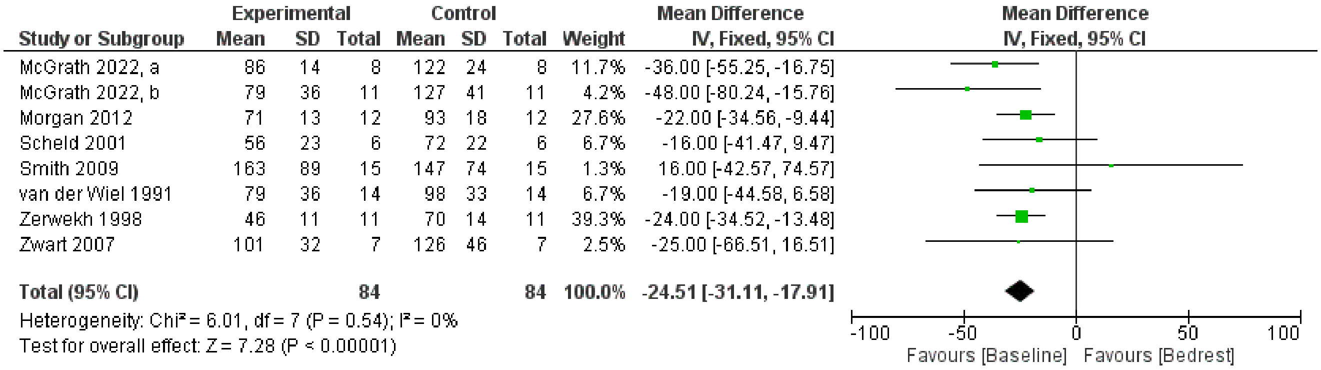

Figure legend: The x-axis indicates the effect on circulating calcitriol in (pmol/l). Negative values indicate a decrease, positive values an increase in circulating calcitriol. Horizontal lines indicate 95% confidence intervals of individual studies

Figure S4B: Funnel plot regarding the effect of bedrest on circulating calcitriol

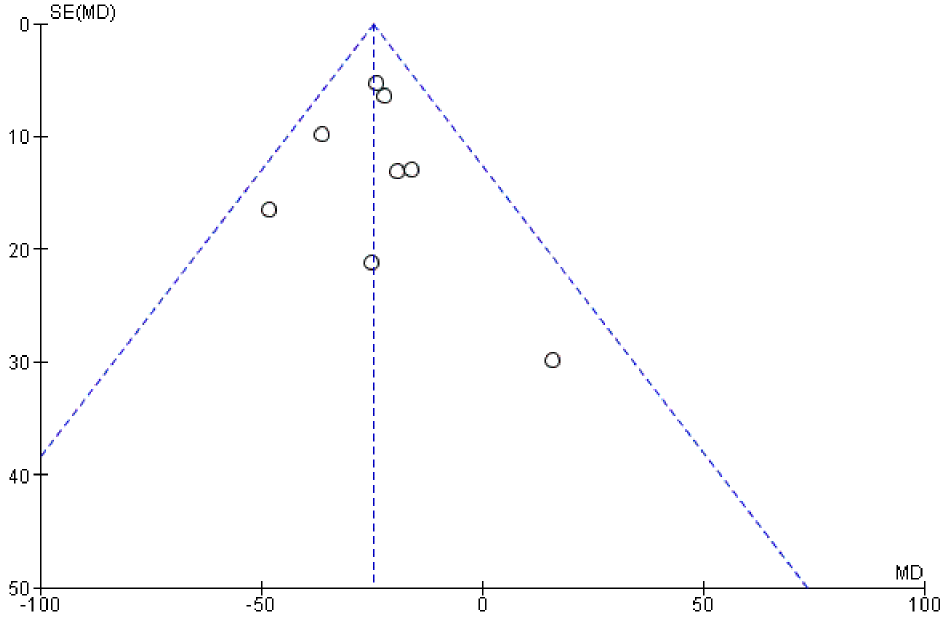

Figure legend: MD indicates mean difference of bedrest vs. control in individual trials; each circle displays the result of an individual study; a value < 0 notifies a decrease in circulating calcitriol and a value > 0 notifies an increase in circulating calcitriol by bedrest; SE (MD) denotes the standard error of the mean difference. Risk of publication bias cannot be ruled out if circles are lying outside the dotted lines.

Figure S5A: Meta-analysis regarding the effect of preeclampsia on circulating calcitriol

Study or Subgroup	Control Mean	Control SD	Control Total	Preeclampsia Mean	Preeclampsia SD	Preeclampsia Total	Weight	Mean Difference IV, Random, 95% CI
August 1992	156	24	9	91	38	9	14.4%	65.00 [35.64, 94.36]
Dalmar 2015	128	60	54	138	62	44	16.2%	-10.00 [-34.32, 14.32]
Halhali 2000	151	31	24	113	24	24	19.2%	38.00 [22.32, 53.68]
Halhali 2007	120	22	26	103	22	26	20.4%	17.00 [5.04, 28.96]
Seely 1992	220	13	24	172	19	12	20.4%	48.00 [36.06, 59.94]
Taufield 1987	146	43	9	101	46	6	9.4%	45.00 [-1.30, 91.30]
**Total (95% CI)**			**146**			**121**	**100.0%**	**32.54 [13.77, 51.31]**

Heterogeneity: $Tau^2 = 413.07$; $Chi^2 = 30.10$, df = 5 (P < 0.0001); $I^2 = 83\%$
Test for overall effect: Z = 3.40 (P = 0.0007)

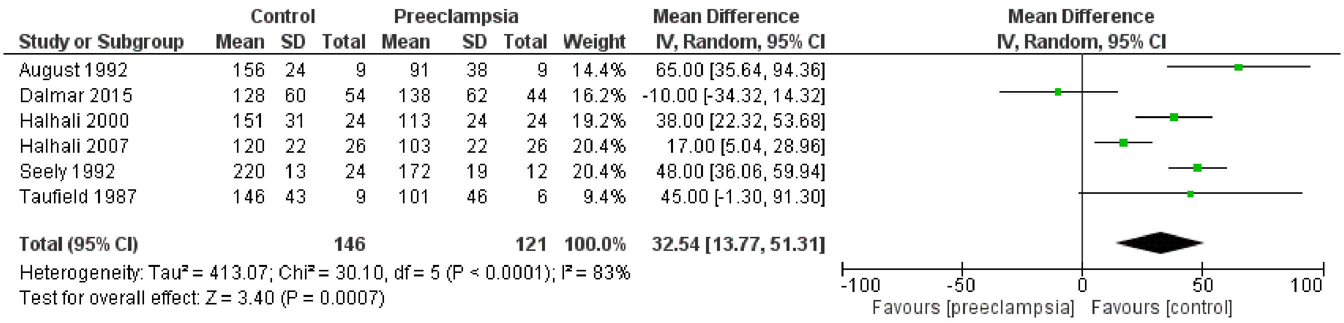

Figure legend: The x-axis indicates the effect on circulating calcitriol in (pmol/l). Negative values indicate a decrease, positive values an increase in circulating calcitriol. Horizontal lines indicate 95% confidence intervals of individual studies

Figure S5B: Funnel plot regarding the effect of preeclampsia on circulating calcitriol

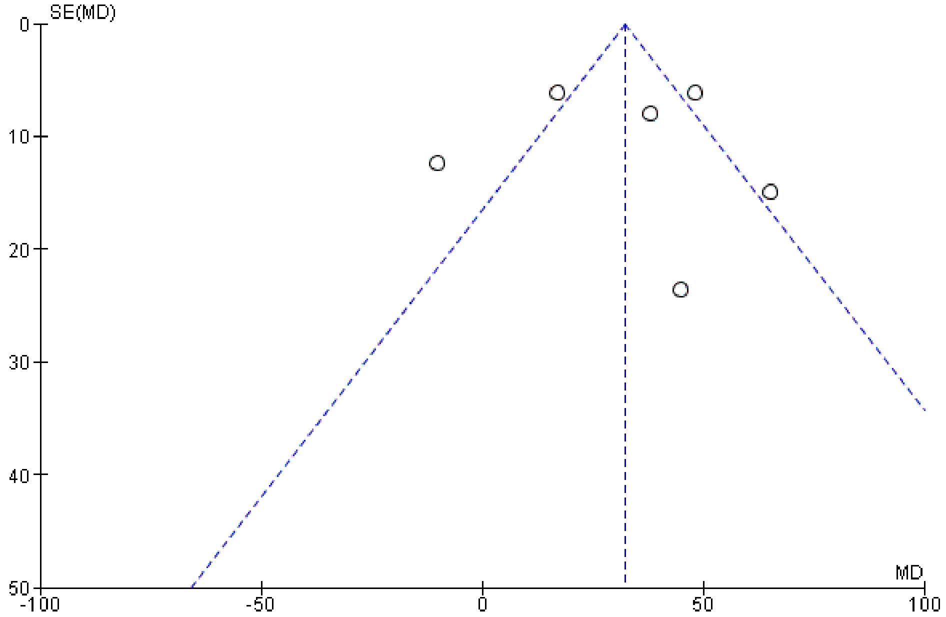

Figure legend: MD indicates mean difference of controls vs. preeclampsia in individual trials; each circle displays the result of an individual study; a value < 0 notifies a decrease in circulating calcitriol and a value > 0 notifies an increase in circulating calcitriol by non-preeclampsia; SE (MD) denotes the standard error of the mean difference. Risk of publication bias cannot be ruled out if circles are lying outside the dotted lines.
